# Workplace health promotion programs for employees in long-term care facilities - a systematic review

**DOI:** 10.1093/eurpub/ckac129.181

**Published:** 2022-10-25

**Authors:** M Herz, D Gebhard

**Affiliations:** TUM Department of Sport and Health Sciences, Technical University of Munich, Munich, Germany

## Abstract

**Background:**

Employees in long-term care facilities (LTC) are exposed to physically and mentally demanding workloads. Due to the specific working conditions and processes in LTC, recent literature recommends that care setting-specific health promotion is required. The objective was to systematically review the current evidence of workplace health promotion programs among employees in LTC.

**Methods:**

A systematic review was conducted in accordance with the PRISMA 2020 Statement. The literature search was applied in the online databases PubMed, Web of Science, Cochrane Central Register of Controlled Trials, and APA PsycArticles (Jan 2000 - Feb 2022). Studies were included if (1) participants worked in any occupational setting in LTC, (2) personal health and outcomes related to occupational health were measured as primary outcome, and (3) studies were randomized controlled trials. Methodological quality was assessed using the Cochrane risk of bias assessment tool (RoB 2).

**Results:**

The literature search yielded 23.007 articles, resulting in 24 included studies and 21 unique interventions with a total of 6.625 participants at baseline. Most participants were female (85.2% to 100%). Interventions were grouped into person-directed (n = 4), person/work interface-directed (n = 10), work-directed (n = 0), and combined approaches (n = 7). Of these studies, two studies (2/4) using a person-directed approach, four studies (4/10) using a person/work interface-directed approach, and four studies (4/7) using a combined approach demonstrated significant improvements in personal health-related outcomes and occupational health-related outcomes. Methodological quality can be rated as some concerns.

**Conclusions:**

Interventions that incorporate a combination of intervention approaches appear promising for improving health and work-related outcomes among employees in LTC. There is a lack of evidence for only work-directed approaches to health promotion in LTC. High-level quality studies are still needed.

**Key messages:**

• Combined intervention approaches to workplace health promotion in long-term care facility settings appear to be beneficial.

• High-level quality studies on workplace health promotion in long-term care facilities are still needed.

